# Improving Motor Skills in Five Children With Developmental Coordination Disorder Traits and Its Impact on Parenting Stress: A Case Series

**DOI:** 10.7759/cureus.61691

**Published:** 2024-06-04

**Authors:** Ryota Hatanaka, Yumi Higuchi, Masakazu Imaoka

**Affiliations:** 1 Rehabilitation, Osaka Kawasaki Rehabilitation University, Kaizuka, JPN; 2 Rehabilitation, Osaka Metropolitan University, Osaka, JPN

**Keywords:** case series, motor-skills training, children, parenting stress, developmental coordination disorder

## Abstract

Poor motor skills in children with developmental coordination disorder (DCD) are associated with childcare stress. This study aimed to assess whether improving the motor skills of children with DCD could reduce parenting stress. The participants were five boys aged 7-10 years with probable DCD and their parents. The intervention comprised 1 hour per week of motor skills training for nine weeks. We measured improvements in the children's motor skills and reductions in parenting stress before and after the intervention. All five children showed improvements in motor skills. Parenting stress was reduced in two parents, whereas it worsened in three parents. Improving motor skills in children with probable DCD may not necessarily reduce parenting stress.

## Introduction

Developmental coordination disorder (DCD) is a condition in which the acquisition and performance of coordinated motor skills are well below age-expected motor levels [1]. The prevalence estimates for DCD have been reported to range between 2 and 20%, with a range of 5-6% being the most cited in the literature. The natural history of DCD did not vary over at least one year of follow-up. Over longer periods, motor skills may improve in children with DCD. However, 50-70% of children with DCD continuously exhibit low motor skills during adolescence [2].

According to the Diagnostic and Statistical Manual of Mental Disorders, Fifth Edition, Text Revision (DSM-5-TR^TM^) definition, the neurodevelopmental disorder group includes attention-deficit/hyperactivity disorder (ADHD), autism spectrum disorder (ASD), specific learning disability, and DCD; among these, DCD is known to have a high comorbidity [2]. Parents of children with ADHD and ASD are highly stressed about childcare [3,4]. In patients with DCD, motor skills are impaired, and acquisition is delayed, severely interfering with activities of daily living and academics [5]. These issues directly increase the frequency and effort required for caring for children, increase opportunities to pay attention to them, and contribute to parenting stress [6]. Children's development is closely related to their relationship with their parents. Parents experiencing high levels of parenting stress and mental instability may be a cause of developing behavioral and social problems in their children. This research suggests that physical therapy may be able to support parents experiencing childcare stress.

Systematic reviews and meta-analyses of physical therapy for DCD have shown that motor-skill training effectively improves motor skills. The effective duration of the intervention was reported to be 10-15 hours. The general principles of motor-skill training are to start with partial practice and build full practice, repeat the task multiple times, have the student perform the task in a variety of environments, train according to progress, promote self-discovery, and provide feedback. It has been reported that group-based interventions were more effective compared with one-on-one interventions, and the placement of assistants in small groups of four to six children was more effective [7]. However, there are no reports on whether the improvement in motor skills in children with DCD traits could reduce parenting stress. Although DCD is rarely diagnosed in Japan, latent cases may still exist [8]. Therefore, DCD in Japan has been reported as a DCD trait.

## Case presentation

Participants

Participants who did not exercise well were recruited from public elementary schools in Osaka Prefecture, Japan, by flyers. Children with neurological disorders that could affect movement (e.g., cerebral palsy) were excluded [2]. We adopted the DSM-5-TRTM criteria. The exclusion criteria were listed on the flyer; diagnoses in children were [A1] established based on interviews. Participants with total test scores below the 16th percentile on the Movement Assessment Battery for Children Second Edition (MABC-2) were considered to have a DCD trait [9]. Based on these criteria, five boys aged 7-10 years were included in the study. One participant was diagnosed with ASD, and four remained undiagnosed. Before motor skill measurements, the parents of the participants were interviewed to confirm that the children were able to follow verbal instructions and that there were no exercise restrictions by their primary care physicians.

Ethics statement

Before obtaining the participants’ consent, all participants and their families were informed in writing and verbally about the purpose and method of the study, the data storage method, and anonymization. This study was approved by the Research Ethics Committee of the Graduate School of Comprehensive Rehabilitation Studies, Osaka Prefecture University (approval number: 2018-117), Japan.

The movement assessment battery for children second edition (MABC-2)

MABC-2 is an international rating scale developed for evaluating children with motor difficulties. All studies were conducted in age band 2 (7 years 0 months to 10 years 11 months). MABC-2 comprises eight items, and the results were converted into standardized scores using the correspondence table. The standardized scores of the eight items were totaled for each of the three components of “manual dexterity”, “aiming and catching”, and “balance”; the ‘total test scores’ were converted into standardized scores using the correspondence table. The standardized scores of the three components were summed to calculate the “total test score” and also converted to a standardized score using a correspondence table.

Currently, the Japanese version of the MABC-2 has not been standardized. Therefore, the correspondence table uses the original British version [9]. Furthermore, previous research has shown that the original British version was highly useful for evaluating Japanese children aged 7-10 years [10]. In addition, using the original British correspondence table, the total test scores were converted to percentiles; the DCD trait was defined as less than the 16th percentile. Minimal clinically important differences (MCID) were used to assess the efficacy of the intervention [11]. The standard error of measurement was defined as the MCID obtained using a distribution-based method. According to the original British manual, the standard errors for the three components ranged between 1.20 and 1.56; the standard error for the total test score should be 1.34. Therefore, all MCIDs were assigned a score of two.

Intervention

The exercise program was devised based on a previous study in which a physiotherapist with 18 years of clinical experience conducted motor-skills training in children with DCD (Table 1) [12]. The exercise duration was 5 min for warm-up exercises, 5 min for dexterity exercises, 15-20 min for task training for throwing and catching movements using rocket-shaped balloons, balloons, and balls, 15-20 min for sitting/standing balance task training, and trunk muscle training; and finally 10-15 min for free tasks. To obtain a positive image of the exercise, we incorporated fun elements into the exercise program. The exercise program was updated every two weeks. If a task was too difficult for a participant during the program, it was adjusted to the participant’s ability by a physiotherapist with extensive experience with children. The exercise program was conducted in a gymnasium at Osaka Kawasaki Rehabilitation University, Japan. Each participant attended a one-hour session weekly, with the exercise program spanning nine weeks.

**Table 1:** Exercise program

	1~2 week	3~4 week	5~6 week	7~9 week
Dexterity movement	Stick a magnet to the whiteboard	Put a sticker on paper	Tear up newspaper	Rock paper scissors
Catching and throwing the ball	Catch a rocket balloon	Throw a balloon into the air and catch it	Throw a crumpled newspaper and catch it	Throw a ball at a target
Balance	Standing on one leg with both hands in front	Standing on one leg with one hand on the side	Dodge a thrown ball	Passing the hula hoop through the body (standing on one leg)
Core stability training	Maintain a standing position against disturbance	Posture fixed with a signal when walking (Red light, Green light Japanese version)	Sit on a physio ball and keep your balance	Crawling race

All participants were assigned an exercise assistant who underwent 5 hours of training. A physiotherapist constantly monitored the performance of all participants. The training comprised 2.5 hours of classroom training on how to play with and talk to children. For the next 2.5 hours, the exercise assistant pretended to be the participant and learned the exercise program. Similar to children's exercise programs, there were breaks every 15-20 minutes. The physiotherapist and exercise assistant held a meeting after each exercise program to share information such as whether the difficulty level of the task was appropriate for the participants, whether the participants enjoyed the exercise, how to adjust the exercise intensity, and how to interact with the participants. To facilitate the introduction of exercise programs for children, the parents were encouraged to participate in exercise programs only after they were introduced.

Parenting stress

Parental stress was measured using the Japanese version of the parenting stress index child domain (PSI-C). The participants' mothers were given a 38-item, seven-subscale stress questionnaire regarding aspects of their children. Each item on the answer sheet was to be answered on a five-point scale from “completely disagree” to “completely agree,” and the respondents were asked to give their honest answers.

Using the PSI-C tally form, the scores of the above five levels were converted into a scale of one to five points and then recorded. Thereafter, each score was totaled and used as the score for “Children’s Aspects.” Aggregated results were converted into percentiles using the “PSI-C Percentile Table.” Percentiles were obtained from the frequency distribution of standard samples, with the standard ranging between 15% and 80%. More than 85% of the participants were judged to have high parenting stress. The MCID was used to evaluate efficacy. Based on the distribution-based method, the standard error of the measurement was defined as the MCID. With reference to the guidance, the standard deviations of the subscale measures ranged between 1.4 and 2.0, and the MCID was determined to be two points each. The scores for children’s aspects had a standard deviation of 4.8, determining an MCID of five points [13].

ASD and ADHD comorbidities assessment

The Social Communication Questionnaire (SCQ) was used to determine the presence or absence of symptoms associated with ASD. There were 40 questions, and the parents of the participating children answered “yes or no.” The SCQ has two types of examination forms, “Birth to present” and “Present.” However, in our study, “From birth to now” was used for screening. Scoring was performed by summing the points to 0 or 1 for each question. According to the Japanese version of the manual, a score of 15 points or more was regarded as an ASD tendency (sensitivity 0.85, specificity 0.75, positive predictive value 0.93, and negative predictive value 0.55) [14].

ADHD rating scale-IV

The home version of the ADHD Rating Scale (ADHD-RS) was given to the parents of the participating children [15]. It has a nine-item Inattention and Hyperactivity-Impulsivity subscale. Three scores were calculated for each (inattention, hyperactivity-impulsivity, and total). The raw score was converted into a percentile value corresponding to the score analysis sheet that matched the gender and age of the participating children. The inattention and hyperactivity-impulsivity cut-off values were set to the 93rd percentile (inattentiveness: sensitivity, 0.83; specificity, 0.49; positive predictive value, 0.58; negative predictive value, 0.81; and hyperactivity-impulsiveness: sensitivity, 0.84; specificity, 0.49; positive predictive value, 0.54; and negative predictive value 0.81) [15].

Physical status: skeletal muscle index (SMI) and obesity 

Height was measured using a stature meter. A body composition analyzer (InBody-270, InBody, Japan) using the bioimpedance method was used to measure the skeletal muscle mass of the limbs, normalized by the square of the height to calculate SMI. Currently, there are no reference values for the SMI in infants in Japan. Therefore, based on data from China, low skeletal muscle mass was defined as below the third percentile value of 3.61 kg/m2 for seven-year-old children and 4.18 kg/m2 for 10-year-old children [16].

The degree of obesity was calculated based on height and weight. In Japan, “the degree of obesity” is used to determine obesity in school-aged children (ages 6-18). The “Pediatric Obesity Clinical Practice Guidelines 2017” define normal as obesity of more than -20% to less than +20%. Therefore, -20% or less was defined as a lean tendency and +20% or more was defined as an obese tendency.

Obesity (%) = 100 × (current body weight - standard body weight)/standard body weight. The standard weight was 0.513 × height (cm) - 38.878 for 7-year-olds and 0.752 × height (cm) - 70.461 for 10-year-olds, based on data from the Ministry of Education, Culture, Sports, Science and Technology's School Health Statistics Survey Report (2000) [17,18].

Data analysis

**Figure 1:**
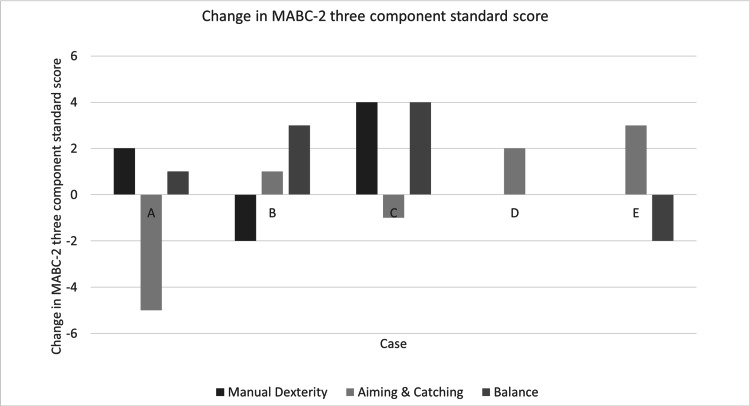
Change in the MABC-2 three-component standard score MCID was defined as a change of two points or more in the standardized score.

**Figure 2:**
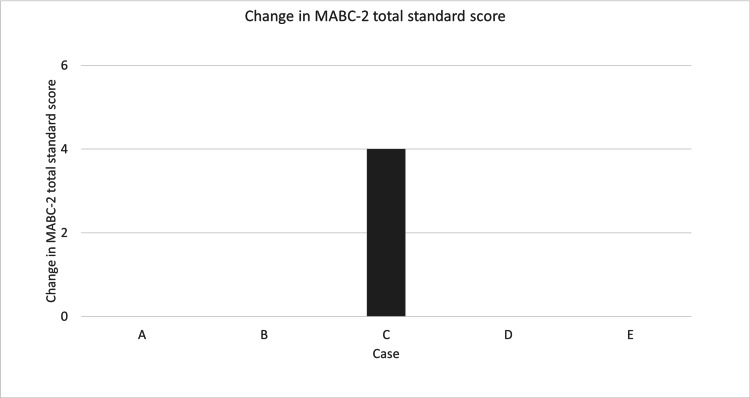
Change in MABC-2 total standard score MCID was defined as a change of two points or more in the standardized score.

**Figure 3:**
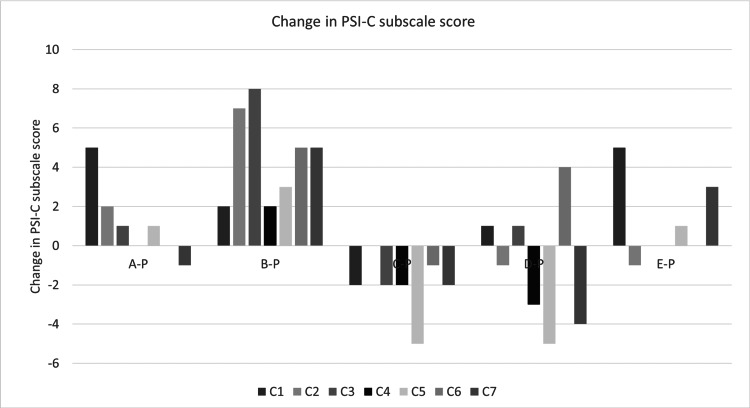
Changes in each item regarding the “child domain” of parenting stress for parents with children with the DCD trait C1: Reinforces Parents, C2: Mood, C3: Acceptability, C4: Distractibility/Hyperactivity, C5: Hang around parents, C6: More problems/worries, C7: Sensitive to stimuli, PCI-C: Parenting Stress Index-Child domain A-P: A parent, B-P: B parent, C-P: C parent, D-P: D parent, E-P: E parent

**Figure 4:**
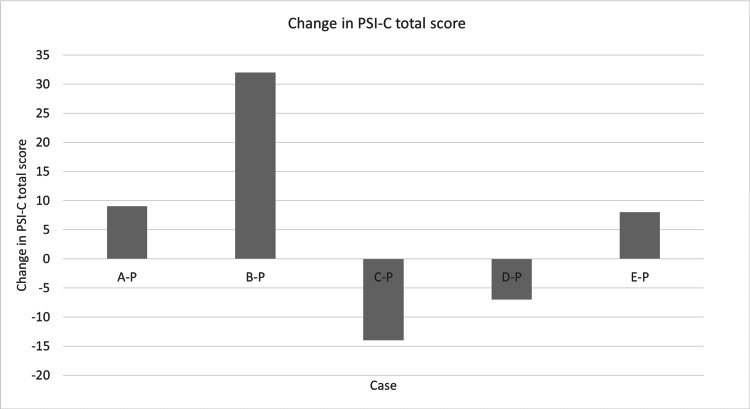
Overall score for child-rearing stress for parents of children with the DCD trait PSI-C: Parenting Stress Index-Child Domain; MCID is defined as a change of five or more points.

Because this was a case series, no statistical analysis was performed. The results for each participant with the DCD trait and their parents are shown in Tables 1-3 and Figures 1-4.

Findings　

Characteristics of Participants With DCD Trait Before Intervention

Table 2 presents age, SMI, degree of obesity, MABC-2, SCQ, and ADHD-RS before intervention. Regarding SMI, participants B, D, and E had low skeletal muscle mass. Considering obesity, all participants with the DCD trait were of normal body type, and none of the participants with the DCD trait were lean or obese. With respect to MABC2, all participating children were below the 16th percentile; all five had the DCD trait. Regarding ADHD-RS, ADHD tendencies were observed in participants B, C, and E. As for SCQA, ASD tendencies were observed only in participant C.

**Table 2:** Baseline characteristics of children with probable developmental coordination disorder (DCD trait) SMI: Skeletal muscle mass index; SCQ: Social communication questionnaire; ADHD-RS: Attention deficit hyperactivity disorder rating scale-IV *1: Low skeletal muscle mass; *2: Suspected ASD; *3: Suspected ADHD

Participant	Age	SMI	Degree of obesity	MABC2 (percentile score)	SCQ	ADHD-RS (percentile score)	Autism spectrum disorder
		(kg/m2)	(%)				
A	7	4	-8.8	5	12	50	
B	7	2.1^＊1^	-16.8	1	13	95^＊3^	
C	7	4.3	16.6	5	26^＊2^	99^＊3^	
D	10	3.6^＊1^	16.5	0.1	12	80	
E	7	3.4^＊1^	-16.1	5	5	96^＊3^	〇

Parenting Stress in Parents With Children With DCD Trait Before Intervention

Table 3 summarizes the parenting stress levels of parents with children with the DCD trait before the intervention. The parents with high parenting stress were C parents (C-P) and D parents (D-P). Regarding the sub-items, there were items in which A parent (A-P), C parent (C-P), D parent (D-P), and E parent (E-P) had high levels of childcare stress. Parenting stress in the child domain was higher for C-P and D-P parents.

**Table 3:** Parenting stress before intervention C1: Reinforces Parents, C2: Mood, C3: Acceptability, C4: Distractibility/Hyperactivity, C5: Hang around parents, C6: More problems/worries, C7: Sensitive to stimuli, A-P: A parent, B-P: B parent, C-P: C parent, D-P: D parent, E-P: E parent ＊: High parenting stress, PSI-C: Parenting Stress Index-Child domain

Participant	C1	C2	C3	C4	C5	C6	C7	PSI-C
	(Percentile)	(Percentile)	(Percentile)	(Percentile)	(Percentile)	(Percentile)	(Percentile)	(Percentile)
A-P	10	30	95^＊^	70	80	99^＊^	95^＊^	80
B-P	10	10	5	1	60	70	80	10
C-P	65	85^＊^	99^＊^	95^＊^	95^＊^	90^＊^	85^＊^	95^＊^
D-P	75	95^＊^	95^＊^	25	95^＊^	99^＊^	99^＊^	95^＊^
E-P	20	70	99^＊^	70	35	95^＊^	15	75

Change in MABC-2 Three Component Scale Score and Total Standard Score

Figure 1 shows the changes in the scores on the MABC-2 three-component scale and the total standard score for the DCD trait. Regarding dexterity, participants A and C showed improvement in their standard scores by two points or more. Participant B’s score worsened by two or more points. Participants D and E showed improvements in aiming and catching, respectively. Only participant A's score worsened. Participants B and C showed improvement in balance, whereas participant E's score deteriorated. Figure 2 illustrates changes in the MABC-2 total standard score for participants with the DCD trait. Only participant C showed an improvement. As this study is a case series, no statistical analysis was performed. However, all participants showed improvements in motor skills. Participant C particularly improved.

Changes in Each Item of Parenting Stress for Parents With Children With DCD Trait

Figure 3 shows the changes in each PSI-C item for parents with children with the DCD trait. The change is the difference between the value before and after the exercise intervention. The bar graph is inverted, with improvement pointing upward and deterioration pointing downward. For “C1: Reinforces Parents,” only the C parent (C-P) score improved by two points or more. Three parents, A parent (A-P), B parent (B-P), and E parent (E-P), had a worsening score of two points or more.

For “C2: mood,” none of the parents of the participating children showed an improvement. Parents A-P and B-P had worsened symptoms. For “C3: Acceptability,” only the C parent (C-P) score improved. However, the B parent (B-P) conditions worsened.

For “C4: Distractibility/Hyperactivity,” C parent (C-P) and D parent (D-P) scores improved, whereas B parent (B-P) condition worsened. For “C5: Hang around parents,” C parent (C-P) and D parent (D-P) scores improved, whereas B parent (B-P) conditions worsened.

No parents improved on “C6: More problems/worries”. Parents B-P and D-P experienced worsening conditions. Parents C-P and D-P improved on “C7: Sensitive to stimuli.” However, parents B-P and E-P had worsening conditions. Three parents (A-P, C-P, and D-P) reported improvements in some items.

Next, describe each individual. Regarding A-P, stress increased in C1 and C2. Regarding B-P, all of C1, C2, C3, C4, C5, C6, and C7 have increased. Regarding C-P, improvements were seen in C1, C3, C4, C5, and C7. Regarding D-P, improvement was seen in C4, C5, and C7, and stress increased in C6. Regarding E-P, stress increased at C4 and C7. Figure 4 shows changes in the PSI-C. The change is the difference between the value before and after the exercise intervention. The bar graph points upward for improvement and downward for deterioration. Two parents, C-P and D-P, improved by five points or more. Three parents, A-P, B-P, and E-P, showed a worsening of five points or more.

Figure 4 shows changes in the PSI-C. The change is the difference between the value before and after the exercise intervention. The bar graph points upward for improvement and downward for deterioration. Two parents, C-P and D-P, improved by five points or more. Three parents, A-P, B-P, and E-P, showed a worsening of five points or more. As this study is a case series, no statistical analysis was performed. However, two parents, C-P and D-P, improved.

## Discussion

Effect of exercise intervention

This study aimed to examine the effectiveness of motor-skill training on the motor skills of children with DCD traits and their parents’ childcare stress. By implementing motor-skill training for the DCD trait, all five children showed improvements in one of the three components. However, the overall score improved for only one out of five participants. Additionally, two parents experienced a reduction in childcare stress after completing their exercise program, whereas three parents experienced worsening stress. The parents of C, whose motor skills had improved, also experienced improvement in childcare stress. Among the four children whose motor skills did not change, the parents of three children, A, B, and E, experienced worsening child-rearing stress, whereas parent D's child-rearing stress improved. These results showed that when motor-skill training was implemented for evaluating children with DCD traits, parents' child-rearing stress could either worsen or be alleviated regardless of the improvement in motor skills.

Effect of training on motor skills

As improvements were observed in all three areas of motor skills through training, the effectiveness of this exercise program was confirmed. However, the reason why four out of five children did not achieve improvements in their overall scores was because the children's families also participated. This approach is different from that recommended in systematic reviews and meta-analyses of physical therapy for children with DCD [7]. For example, the participating child may have higher exercise ability compared with their family members, and the amount of exercise may not have been sufficient for the participating child.

Effect of exercise intervention on parenting stress

In a study of 5-12-year-olds with DCD, Jijon et al. found that parenting stress was associated with poorer motor skills in children and was an important predictive factor; however, it was not a significant predictor [19]. Furthermore, they concluded that children’s behavioral problems are important predictors. In this study, the children may have comorbidities such as ASD or ADHD. Therefore, consideration of their behavioral problems is necessary. Previous research has reported that child-rearing stress in the parents of children with ADHD is reduced by the child’s physical activity [20]. This is explained by an improvement in the child’s self-regulation ability, which is a symptom of ADHD, through physical activity. In this case, participant C had ADHD tendencies, and it is possible that the exercise program improved his self-regulation ability, which became noticeable in his behavior. In addition, in child D, whose parents’ childcare stress was reduced, no behavioral problems such as ASD or ADHD were observed.

Limitations

A systematic review and meta-analysis of physical therapy for DCD reported that the duration of intervention was 10 to 15 hours. Although the intervention period this time was 9 hours, a trend toward improvement was observed. However, since motor skills have not returned to the level of a typically developing child, we believe it is necessary to verify if the child's motor skills have improved to the level of a typically developing child. Also, this study did not examine whether child-rearing stress is due to parents' problems or whether it is due to social (economic, etc.) factors. Since this study included five cases, further research with a statistically appropriate number of cases in the future is warranted. Furthermore, the study design also needs to be validated by an RCT design.

## Conclusions

In this study, motor skill training was performed on the DCD trait, and some motor skills improved, and some did not. In addition, some cases of child-domain parenting stress were reduced or exacerbated. The take-home message from this research is that DCD traits should be examined not only for motor skills training but also for parental approaches to reduce childcare stress. In DCD, parenting stress can be influenced not only by motor skills but also by a variety of other factors. It may also be necessary to provide not only motor skills training for children with DCD but also approaches for parents.
